# Harmonic Generation Microscopy 2.0: New Tricks Empowering Intravital Imaging for Neuroscience

**DOI:** 10.3389/fmolb.2019.00099

**Published:** 2019-10-09

**Authors:** Hyungsik Lim

**Affiliations:** Department of Physics and Astronomy, Hunter College and the Graduate Center of the City University of New York, New York, NY, United States

**Keywords:** intravital imaging, second-harmonic generation, third-harmonic generation, non-linear optical microscopy, label-free imaging

## Abstract

Optical harmonic generation, e.g., second- (SHG) and third-harmonic generation (THG), provides intrinsic contrasts for three-dimensional intravital microscopy. Contrary to two-photon excited fluorescence (TPEF), however, they have found relatively specialized applications, such as imaging collagenous and non-specific tissues, respectively. Here we review recent advances that broaden the capacity of SHG and THG for imaging the central nervous system in particular. The fundamental contrast mechanisms are reviewed as they encode novel information including molecular origin, spectroscopy, functional probes, and image analysis, which lay foundations for promising future applications in neuroscience.

## Introduction

Our knowledge is inherently limited for a biological process lacking an experimental replica, e.g., *ex vivo* cultured cells or *in vitro* reconstitution. Even when such a system exists, questions still linger whether it recapitulates *in vivo* phenomena properly so that we can accept the data for inductive reasoning. Visualizing native tissue is therefore crucial for unraveling the complex dynamics of health and disease. Light microscopy is the method of choice for intravital studies of small animals. Combining non-invasiveness and ~1 μm resolution, it is irreplaceable for studying cellular dynamics. The field of view of ~1 mm^2^ and the imaging depth up to ~1 mm achievable via non-linear optical excitation are ideal for visualizing model embryos and whole organisms.

Historically, second-harmonic generation (SHG) was the first non-linear optical process to be observed experimentally (Franken et al., 1961) and demonstrated for microscopy (Hellwart and Christen, 1974; Sheppard et al., 1977; Gannaway and Sheppard, 1978), which predates the invention of two-photon excited fluorescence (TPEF) microscopy (Denk et al., 1990). Along with the TPEF technique, scanning laser microscopy of SHG and third-harmonic generation (THG) matured in the 1990's when practical mode-locked lasers emerged (Barad et al., 1997; Guo et al., 1997; Gauderon et al., 1998; Muller et al., 1998; Squier et al., 1998; Campagnola et al., 1999, 2002; Millard et al., 1999; Yelin and Silberberg, 1999; Moreaux et al., 2000). Excellent reviews exist in the literature on the basic theory and practice of harmonic generation microscopy (Moreaux et al., 2000; Campagnola and Loew, 2003; Millard et al., 2003; Sun et al., 2004; Debarre et al., 2006; Weigelin et al., 2016; Mostaco-Guidolin et al., 2017).

Here we review optical harmonic generation for intravital imaging in neuroscience. The goal is to explore the “killer applications” of SHG and THG that are hard to replicate with other imaging modalities. Compared to TPEF which is widely used for hypothesis testing, harmonic generation microscopy has been applied in relatively specialized areas, despite a longer history and common instrumentation. Any successful imaging technique must offer new information whose value exceeds any additional cost and complexity. Indisputably, the most outstanding capability of non-linear optical microscopy, TPEF and SHG/THG alike, is to image live cells in deep tissue with low phototoxicity (Centonze and White, 1998; Squirrell et al., 1999; Helmchen and Denk, 2005). Recently developed tricks of optical harmonic generation are surveyed with a special focus on imaging the central nervous system (CNS) where unique contrast mechanisms provide the foundation for killer applications to make novel discoveries.

## Peptide Bonds as a Source of Endogenous SHG

Let us address the molecular origin and supramolecular phase engendering protein SHG which are key to the interpretation of SHG data. The second-order non-linear polarization underlying SHG is a coherent superposition of hyper-Rayleigh scattering (HRS) from single dipole moments, i.e., harmophores.

(1) $$\begin{array}{l} P_{i}\left(2\omega \right)=\sum_{j=1}^{3}\sum_{k=1}^{3}\chi_{ijk}E_{j}\left(\omega \right)E_{k}\left(\omega \right)\\ \;=\sum_{j=1}^{3}\sum_{k=1}^{3}\int \beta_{ijk}E_{j}\left(\omega \right)E_{k}\left(\omega \right)\;dv \end{array}$$

A material's contribution to SHG is characterized by the macroscopic susceptibility χ_*ijk*_, which is a sum of the microscopic hyperpolarizabilities β_*ijk*_ of an ensemble of harmophores within the coherence volume of the excitation beam. It is well-known that an organic compound with a large asymmetric charge transfer (the “push-pull chromophore”) has a substantial hyperpolarizability (Chemla and Zyss, 1987). Nonetheless, the molecular origin of biological SHG remained unclear until it had been determined, by sum frequency vibrational spectroscopy (Rocha-Mendoza et al., 2007) and also indirectly by HRS measurements (Deniset-Besseau et al., 2009), that the delocalized π-electron between –C = O and –N-H of the peptide (or amide) bond is primarily responsible for SHG from collagen. This finding is also consistent with the experimental observation that SHG signal is relatively independent of the specific sequence of collagen, Gly-X-Y. A remarkable corollary of this is that a much larger group of peptides and proteins, either synthetic or gene-expressed, are potentially SHG-active than previously assumed (Duboisset et al., 2013). It has tremendous implications for intravital SHG microscopy.

Most proteins are not as SHG-active as type 1 collagen, despite containing the same peptide bonds. Non-centrosymmetry is often cited as a necessary condition for non-vanishing HRS or SHG, but for large biological macromolecules it is the rule rather than an exception. For the second-order susceptibility, the supramolecular organization of harmophores is as much essential as the chemical composition determining the hyperpolarizability. An additional requirement on the spatial ordering of harmophores is illustrated below. For a simple example of uniformly oriented uniaxial molecules, Equation (1) becomes

(2) $$P_{i}\left(2\omega \right)=\frac{N}{V}\beta \;\sum_{j=1}^{3}\sum_{k=1}^{3}E_{j}\left(\omega \right)E_{k}\left(\omega \right)$$

(3) $$I_{SHG}\propto {\left|P_{i}\left(2\omega \right)\right|}^{2}\sim {\left(\frac{N}{V}\right)}^{2}{\left|\beta \right|}^{2}I_{Ex}^{2}$$

The SHG intensity is proportional to the density of harmophores squared in contrast to TPEF which is proportional to the density of chromophores. Consequently, even from molecules with HRS weaker than TPEF by ~4 to 10 orders of magnitude (Moreaux et al., 2000, 2001), SHG signal can be significant provided they are abundant and aligned uniformly. The sensitivity of SHG to the spatial ordering of harmophores makes it possible to treat a structural protein as an ensemble of peptide bonds organized in a phase, analogous to a concept in condensed-matter physics. Then the structural alteration of the protein in health and disease may be considered as a phase transition.

### Microtubules and Neurodegeneration

**Figure 1:**
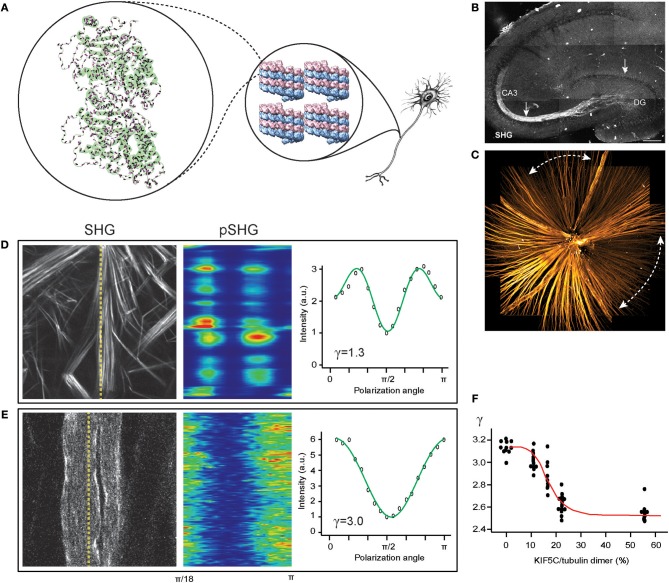
SHG for imaging the central nervous system (CNS). **(A)** Tubulin heterodimer (PDB: 1TUB) with peptide bonds (magenta rods) in microtubule lattice (PDB: 5SYF), which are uniformly oriented in the axon. **(B)** The mossy fibers in an acute slice of the hippocampus imaged by SHG (reproduced from Dombeck et al., 2003). **(C)** The retinal nerve fiber bundles in the fresh wholemount retina of glaucoma-developing DBA mouse visualized by SHG. Dashed lines, characteristic sectorial degeneration of glaucoma (redrawn from Sharoukhov et al., 2018). **(D,E)** SHG intensity for collagen **(D)** and microtubules **(E)**, respectively, and SHG polarization anisotropy (pSHG) corresponding to the dashed lines as a function of the polarization angle (redrawn from Sharoukhov and Lim, 2016). **(F)** SHG anisotropy of microtubule changes upon KIF5C binding. Red line, the best fit to the modified Hill equation (redrawn from Shima et al., 2018).

Collagen fibrils are a well-known example representing an extracellular pseudocrystalline phase studied by SHG (Roth and Freund, 1979, 1982; Freund et al., 1986). By contrast, an intracellular phase is found in the cytoskeletal element of microtubules from which SHG has been detected in the context of mitotic spindles (Campagnola et al., 2002; Bancelin et al., 2017) and axons (Dombeck et al., 2003; Kwan et al., 2008; Lim and Danias, 2012a; Psilodimitrakopoulos et al., 2012; Van Steenbergen et al., 2019). The unique phase of microtubules, i.e., uniform polarity, is a defining characteristic of axons as opposed to dendrites (Baas et al., 1988), rendering the SHG signal specific to the axons (Figures 1A–C). Furthermore, the long persistence length of microtubules, ~1 mm (van den Heuvel et al., 2007; van Mameren et al., 2009), compared to that of collagen fibril, ~4 μm (Varma et al., 2016), is a factor for increasing the order of harmophores within the focal volume.

SHG as a neuroimaging modality offers new opportunities for investigating neurodegenerative disorders, such as Alzheimer's disease and glaucoma, where disruption of axonal microtubules has been implicated (Matsuyama and Jarvik, 1989; Cash et al., 2003; Huang and Knighton, 2005; Huang et al., 2006; Zempel et al., 2010). The pathogenic processes have been visualized by microtubule SHG, e.g., lesions near senile plaques in a mouse model of Alzheimer's disease (Kwan et al., 2009), tau-tubulin interaction (Stoothoff et al., 2008), and neuronal ischemia (Psilodimitrakopoulos et al., 2013). In our own lab, we have utilized SHG from the retinal nerve fiber bundles (i.e., the axons of the retinal ganglion cells) to obtain new findings on the glaucoma pathogenesis (Figure 1C); specifically, we found that the disruption of pseudocrystalline microtubules occurs earlier than the loss of the retinal nerve fiber bundles and that they are likely to share a common pathogenic insult (Lim and Danias, 2012a,b; Sharoukhov et al., 2018).

### Conformational Dynamics and Allostery *in vivo*

The notion to view a protein macromolecule as a phase of peptide bonds is the basis of a new SHG trick, i.e., to measure the conformational dynamics in a physiological tissue. Allosteric changes upon biochemical cues as they occur in the native microenvironment, although fundamental for understanding biological functions, cannot be captured by the traditional methods of structural biology involving fixation of the specimen, nor by light microscopy whose diffraction-limited resolution is beyond the size of macromolecules. SHG can, by virtue of coherent superposition, encode conformational features which can help deduce allosteric modifications. For example, the average angle, φ, between the molecular orientation and the symmetry axis can be deduced from the experimentally-determined second-order susceptibility (Roth and Freund, 1979; Heinz et al., 1983; Dick, 1985; Freund et al., 1986; Mazely and Hetherington, 1987; Simpson and Rowlen, 1999; Kriech and Conboy, 2003). Briefly, the susceptibility tensor in Equation (1) is given as a sum of the hyperpolarizabilities of peptide bonds weighted with the direction cosines of the relative angles.

(4) $$\chi_{ijk}=\sum_{i^{\prime }j^{\prime }k^{\prime }}\langle \cos\varphi_{ii^{\prime }}\cos\varphi_{jj^{\prime }}\cos\varphi_{kk^{\prime }}\rangle \beta_{i^{\prime }j^{\prime }k^{\prime }}$$

For the simplest form of harmophore with *C*_∞_ symmetry, whose hyperpolarizability tensor β_*ijk*_ is dominated by a single element, there are only four independent and non-vanishing susceptibility tensor elements, i.e., χ_*xxz*_, χ_*zxx*_, χ_*zzz*_, and χ_*xyz*_, where z is the symmetry axis. In the case of off-resonant excitation for which the indices are permutable (i.e., the Kleinmann symmetry), only two of them are independent and non-vanishing - χ_*zxx*_ and χ_*zzz*_. Then the ratio is related to the average polar angle, φ, of harmophores relative to the symmetry axis.

(5) $$\frac{\chi_{zzz}}{\chi_{zzz}+2\chi_{zxx}}\;=\;\frac{\gamma }{\gamma +2}\;=\frac{\langle \cos^{3}\;\varphi \rangle }{\langle \cos\;\varphi \;\rangle }≅\cos^{2}\;\langle \varphi \rangle ,\gamma \equiv \frac{\chi_{zzz}}{\chi_{zxx}}\;$$

As a result, the average orientation of harmophores can be obtained regardless of the details of the hyperpolarizability. Experimentally, γ can be determined by measuring the SHG intensity as the linear polarization of the excitation is rotated at an angle θ relative to the symmetry axis.

(6) $$I_{SHG}\propto \sum_{i}{\left|P_{i}\left(2\omega \right)\right|}^{2}\sim E{\left(\omega \right)}^{4}\;\left\{{\left(\sin^{2}\theta +\gamma \;\cos^{2}\theta \right)}^{2}+\sin^{2}\;2\theta \right\}$$

The measured ratio can then be compared with the predicted value from the Protein Data Bank (PDB). This method is referred to as SHG polarization anisotropy or polarization-resolved SHG (pSHG).

SHG polarization anisotropy has been demonstrated for collagen (Roth and Freund, 1979, 1982; Freund et al., 1986; Stoller et al., 2002, 2003; Chu et al., 2004; Su et al., 2011) and myosin (Boulesteix et al., 2004; Plotnikov et al., 2006; Chu et al., 2007; Tiaho et al., 2007; Odin et al., 2008). Remarkably, the pSHG analysis of collagen has distinguished the isoforms (Su et al., 2010; Tilbury et al., 2014), which cannot be done by antibodies, and also measured deformations under mechanical stress (Gusachenko et al., 2012; Mansfield et al., 2019). Similarly, the pSHG analysis of myosin has resolved distinct physiological states during muscle movement (Nucciotti et al., 2010). SHG polarization anisotropy is a powerful technique to detect the relative magnitude of the hyperpolarizability of chemical moiety, which is otherwise difficult to measure. For instance, the effect of the methylene groups in proline and hydroxyproline amino acids accounted for the deviations of the measured pSHG from the theoretical predictions (Rocha-Mendoza et al., 2007; Su et al., 2011). However, artifacts could hamper the correct interpretation of pSHG data; for a heterogeneous distribution of polar angles, the measured average polar angle has an apparent value of ~39.2° which is known as the SHG magic angle (Simpson and Rowlen, 1999).

SHG polarization anisotropy has been demonstrated for axonal microtubules in the CNS. The conformation of microtubules underlies the regulation mechanisms by ligands and microtubule-associated proteins (MAPs). Straight GTP-bound tubulins are readily incorporated into microtubule lattices whereas the mechanical strain produced by curved GDP-bound tubulins can lead to a catastrophic breakdown (Wang and Nogales, 2005) and switching from straight to curved conformations upon GTP hydrolysis promotes the disassembly of microtubules (Muller-Reichert et al., 1998) while binding with the anti-mitotic agent paclitaxel (or taxol) induces a straight conformation to stabilize the microtubule even with GDP-bound tubulins (Elie-Caille et al., 2007). We tested pSHG for measuring allosteric dynamics upon binding with taxol. The pSHG profile of microtubules was much different from that of collagen (Figures 1D,E) (Odin et al., 2009; Psilodimitrakopoulos et al., 2012, 2013; Sharoukhov and Lim, 2016), but the overall effect of taxol was too small to resolve (Sharoukhov and Lim, 2016). Recently, the conformational change upon binding to kinesin KIF5C has been investigated using pSHG and microtubules *ex vivo* from *Xenopus laevis* egg extract, and the data suggests considerable allostery of microtubules (Shima et al., 2018) (Figure 1F). Another promising neurological field of research for SHG polarization anisotropy is brain traumatic injury. While it remains elusive how mechanical strain induces pathogenesis and neurodegeneration (Tang-Schomer et al., 2010; Johnson et al., 2013; Hill et al., 2016), the microtubule cytoskeleton, which is largely responsible for the mechanical property of the axon, is presumed to play a significant role. It is anticipated that many discoveries of the biomechanics of axon injury will be achieved with pSHG.

### Decoding SHG by Machine Learning

In addition to developing SHG readouts encoding molecular characteristics, innovations have been achieved for decoding the information. Phase transition is a collective phenomenon on a macroscopic scale beyond the individual pixels; therefore, it is conceivable that the microdomains of structural proteins can be defined more accurately via inter-pixel relationships. A famous method to extract quantities across the neighboring pixels is texture analysis, in which a set of first- and second-order image statistics are evaluated from the gray level co-occurrence matrix (GLCM) (Haralick et al., 1973). Following the earlier applications to medical imaging, texture features have been extracted from SHG images from which subtle differences could be identified that are difficult to detect by human vision (Nadiarnykh et al., 2010; Adur et al., 2011; Watson et al., 2012; Mostaco-Guidolin et al., 2013; Wen et al., 2014, 2016). A particularly promising application of SHG texture analysis appears to be the classification of the extracellular matrix (ECM). Remodeling of the ECM in the tumor microenvironment has been recognized as a potential biomarker, providing an orthogonal basis to conventional histology based on cellular morphology (Provenzano et al., 2006, 2008). SHG textures of the ECM have been demonstrated for machine-aided evaluations of pathology (Watson et al., 2012; Mostaco-Guidolin et al., 2013; Wen et al., 2016; Xu et al., 2016). Similarly, it is expected that SHG textures will yield useful information about the CNS tissues. For the purpose of image classification, textures need not be associated with biophysical parameters (e.g., the persistence length), but such a priori knowledge would be critical for modeling the molecular basis of microtubule biomechanics and the interpretation of SHG data.

## Exogenous Molecules for Functional SHG Imaging

One of the most important applications of live-cell SHG imaging has been the studies of membranes, where the lipid bilayer provides a substrate for spatially ordered harmophores. On account of a smaller number of harmophores that can be incorporated into the plasma membrane (micromolar, or ~1 per 100 lipids) than in structural proteins (millimolar) the hyperpolarizability of individual harmophores becomes crucial for measureable SHG signals. Organic compounds have been employed as exogenous agents for SHG contrast, e.g., styryl dyes (Huang et al., 1988; Bouevitch et al., 1993; Ben-Oren et al., 1996) and all-trans retinals (Nemet et al., 2004; Theer et al., 2011), whose hyperpolarizabilities have been further increased via molecular engineering (Reeve et al., 2010) and/or resonant enhancement. They are designed to be adsorbed in one leaflet of the lipid bilayer for uniform polarity and diffusion to the other leaflet leads to the loss of SHG signals (Srivastava and Eisenthal, 1998). The sensitivity to harmophores' orientation facilitates interesting applications of SHG, e.g., for measuring the time courses of flip-flop (Moreaux et al., 2001) and exocytosis (Millard et al., 2005). Exogenous probes are of particular interest for functional SHG imaging in neuroscience as described below.

### SHG Voltage-Sensors

A main goal of intravital imaging of the brain is the spatiotemporal mapping of action potentials during cognitive activities. Membrane-targeting exogenous harmophores facilitate the measurement of depolarization events by SHG (Huang et al., 1988; Bouevitch et al., 1993; Ben-Oren et al., 1996; Dombeck et al., 2004; Nemet et al., 2004; Nuriya et al., 2006; Sacconi et al., 2006; Jiang et al., 2007; Teisseyre et al., 2007; Theer et al., 2011). The mechanism of voltage-dependent SHG stems from the third-order non-linear polarization.

(7) $$\begin{array}{l} P\left(2\omega \right)=\chi^{\left(2\right)}E\left(\omega \right)E\left(\omega \right)+\chi^{\left(3\right)}E_{m}E\left(\omega \right)E\left(\omega \right)\\ \;=\left(\chi^{\left(2\right)}+\chi^{\left(3\right)}E_{m}\right)E\left(\omega \right)E\left(\omega \right) \end{array}$$

Ordinarily, the **χ**^**(3)**^ SHG effect is negligible, with the relative magnitude of the second- to third-order susceptibilities in the order of the atomic field strength (χ^(2)^/χ^(3)^~10^9^ to 10^11^ V/m) (Boyd, 1999). However, the electric field across a membrane during depolarization is a considerable fraction of this (*E*_*m*_~10^7^ V/m). SHG voltage-sensors have a number of advantages over fluorescent probes: Since the voltage-sensitive SHG is mainly an electrooptic effect occurring without significant molecular rearrangements (Jiang et al., 2007), it can trace the rapid rise and fall of action potentials (Pons et al., 2003; Jiang et al., 2007; Theer et al., 2011). Also, because SHG does not involve the absorption of excitation energy leading to photodamage pathways, it is possible to interrogate neuronal activities for an extended period.

However, SHG imaging of neuron firing in the living brain is a challenge. Despite the use of customized SHG molecules, the signal-to-noise ratio (SNR) is still low compared to fluorephores and many line-scans must be averaged to obtain a reasonable SNR. The susceptibility can be enhanced by means of near-resonance excitation at the expense of low background and the safety of the illumination. Consequently, reducing photodamage is key to the success of the voltage-sensing SHG technique (Sacconi et al., 2006). An intravital setting requires the forward-propagating SHG radiation to be collected by epidetection after multiple scattering (Beaurepaire and Mertz, 2002) which can be even less efficient than the forward-detection as previously employed for cultured cells and acute slices (Dombeck et al., 2005). For more practical intravital applications, efforts continue to improve SHG voltage-sensors, including porphyrin whose extended π-conjugated system affords 5-10 times larger voltage-dependent susceptibilities than styryl dyes (Reeve et al., 2013) and non-fluorescent SHG dye suitable for multimodal imaging (Nuriya et al., 2016). The voltage sensitivity of styryl dyes has been shown to depend on the non-chromophore (Millard et al., 2004), which implies that it may be possible to optimize the χ^(**3**)^ SHG component independently from the χ^(**2**)^ SHG.

### Genetically-Encoded SHG Probes

The success of TPEF microscopy is much indebted to genetically-encoded probes for labeling specific molecules and cells, e.g., green fluorescent protein (GFP). With the peptide bonds identified as SHG harmophores, it seems plausible to engineer genetically-encoded SHG probes. Synthetic peptides have been designed as SHG probes for studying various dynamics such as membrane penetration (Rao et al., 2014) and amyloidosis (Ni et al., 2019). Interestingly, the cyclic tripeptide chromophore of GFP, which exhibits an unusually large intramolecular charge transfer (de Meulenaere et al., 2009), has been recognized as a source of SHG (Lewis et al., 1999; Khatchatouriants et al., 2000; Asselberghs et al., 2008). The hyperpolarizability of GFP-like proteins has been optimized via directed evolution, achieving two- to three-fold enhancement (Remington et al., 2005; de Meulenaere et al., 2013). Genetically-encoded SHG probes, either peptide- or GFP-based, would be beneficial for functional imaging of the CNS by allowing the firing of specific cell types of interest to be monitored in living tissue—a formidable task for organic dyes. A variety of genetically-encoded fluorescent sensors, including Ca^2+^ sensor GCaMP, have been developed for probing neuronal activities (Tian et al., 2009; Chen et al., 2013; Fosque et al., 2015; Lin and Schnitzer, 2016; Dana et al., 2019). However, the direct measurement of action potentials in live animals is still immature (Peterka et al., 2011; Bando et al., 2019). The SHG intensity from GFP is found to modulate with depolarization (Khatchatouriants et al., 2000). Also, fluorescent proteins have been designed to align relative to the membrane by incorporating a membrane-localization motif of K-Ras4B, although the resultant molecule exhibited little voltage-dependent SHG (Jinno et al., 2014). In these previous experiments, the cyclic tripeptide chromophore of the GFP mutants were not embedded inside the transmembrane space, but it has been argued that the charge redistribution in the surface of the β barrel can cause the GFP chromophore to respond to membrane potentials (Khatchatouriants et al., 2000). Alternatively, it is conceivable that transmembrane proteins with a large voltage-sensitive hyperpolarizability can be rationally designed with an inspiration from natural mechanotransducers (Coste et al., 2010, 2012).

## THG for 3D Edge-Detection

**Figure 2:**
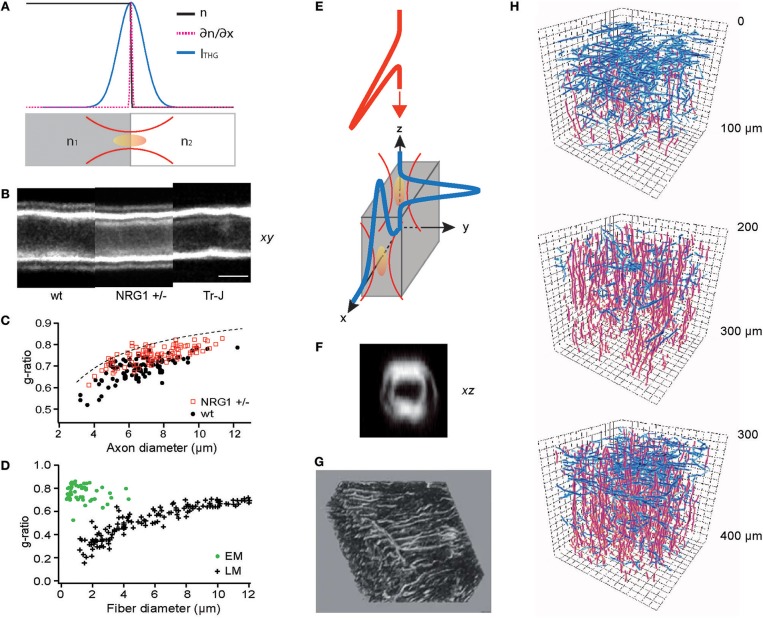
THG for optical metrology and 3D reconstruction of connected meshworks. **(A)** THG as an optical edge detection, as illustrated in Equation (8). **(B)** THG images of the sciatic nerves of wild-type (WT), haploinsufficient NRG1 knockout (NRG1 +/–), and trembler mutant (Tr-J) mice. **(C)** The g-ratios evaluated for WT and NRG1 +/– mice (*N* = 4 each) (reproduced from Lim et al., 2014). **(D)** The g-ratio evaluation by electron (EM) and light microscopy (LM) (redrawn from Waxman and Bennett, 1972). **(E)** Surfaces perpendicular or parallel to the optic axis can be detected by THG. **(F)** An axial section of the myelinated sciatic nerve showing the entire circumference (reproduced from Lim et al., 2014). **(G)** A 3D rendering of the lacuno-canalicular network (LCN) visualized by THG (adapted from Genthial et al., 2017). **(H)** Traces of cortical myelin imaged by THG, horizontal (blue) and vertical (red) fibers in three depths (approximately in layers 1, 2/3, and 4, respectively), showing the distinct abundance of horizontal fibers in layers 1 and 4 (redrawn from Redlich and Lim, 2019).

THG has been applied for label-free imaging of the brain (Farrar et al., 2011; Witte et al., 2011), employing an excitation wavelength longer than the typical output of a mode-locked Ti:Sapphire laser. The additional cost in instrumentation is justified by the unique information provided by the contrast. On account of the Gouy phase shift within a tight focus of the excitation beam, THG destructively interferes within a homogenous bulk medium except in the vicinity of an interface (Ward and New, 1969; Tsang, 1995; Barad et al., 1997). The numerically evaluated intensity of THG (Cheng and Xie, 2002; Carrasco et al., 2006; Olivier and Beaurepaire, 2008) indicates that the image formation can be approximated as an incoherent process in which the magnitude of the 3D gradient of the refractive index *n* is convolved with the point spread function (PSF) of the three-photon excitation, *h*(*x, y, z*) (Figure 2A).

(8) $$\begin{array}{l} I_{THG}\sim h\left(x,y,z\right)⊗\left\|\chi^{\left(3\right)}\cdot \nabla n\left(x,y,z\right)\right\|\\ \sim h⊗\sqrt{{\left(\frac{\partial n}{\partial x}\right)}^{2}+{\left(\frac{\partial n}{\partial y}\right)}^{2}+{\left(\frac{\partial n}{\partial z}\right)}^{2}} \end{array}$$

Notably, gradient magnitude is a well-known operator of edge detection in image processing (Marr and Hildreth, 1980), suggesting that the THG process performs an *optical* edge detection. Below are a few selected applications following this insight.

### Precise Optical Metrology

The myelin sheath is an insulating layer around axons in vertebrates facilitating faster conduction of action potentials. Although crucial for the diagnosis and research into numerous neurological diseases including multiple sclerosis (MS) and schizophrenia, the morphology of individual myelinated fibers in living animals cannot be determined by current clinical modalities, e.g., magnetic resonance imaging (MRI). Furthermore, the standard procedure for measuring the ratio of axon to fiber diameter (the g-ratio), which is a principal biometric for the conduction speed, is prohibitively laborious and time-consuming (~days), involving fixation, imbedding, sectioning, and electron microscopy (EM). Various methods of optical metrology have been developed to address this issue (Kwon et al., 2017). In particular, THG morphometry exploits the image contrast's ability to precisely locate the boundaries between water and lipids (Lim et al., 2014) (Figures 2B,C), rapidly (~30 min) evaluating the g-ratio within a large volume of intact tissue. By contrast, acquiring EM sections over 1-mm^3^ nerve tissue with a sampling voxel of 10^3^ nm^3^ at a typical 1-MHz sampling rate will take as much as 10^9^ s (or ~32 years). Optical metrology of the myelin sheath is not without shortcomings. While sufficiently accurate to distinguish the subtle difference between wild-type (WT) and haploinsufficient NRG1 knockout (NRG1 +/–) mice, the precision of THG morphometry is limited when the spacing between the adaxonal and abaxonal membranes is much smaller than the wavelength, leading to a sampling bias (Figure 2C). The g-ratios evaluated by light microscopy (LM) and EM reveal different types of biases (Sanders, 1948; Waxman and Pappas, 1971; Waxman and Bennett, 1972) (Figure 2D). However, it is worthwhile to note that THG morphometry depends on the centroids of intensity profiles so the precision may be improved beyond the diffraction-limited optical resolution (Bobroff, 1986) as long as the approximation in Equation (8) remains valid.

### 3D Reconstruction of Acicular Networks

THG has been adopted to visualize many cell types and tissues (Aptel et al., 2010; Olivier et al., 2010; Lo Celso et al., 2011; Witte et al., 2011; Small et al., 2018) which can be also imaged by reflectance-based techniques such as optical coherence tomography (OCT). A point differentiating THG from reflectance is illustrated in Equation (8); contrary to reflectance occurring only at the surfaces perpendicular to the optic axis, THG irradiates regardless of the orientation (Figure 2E). This property allows the acquisition of the full 3D morphology, for instance, of the myelinated fibers in living tissue (Figure 2F). Recently, intravital THG imaging has been demonstrated to visualize in the calvaria the lacuno-canalicular network (LCN), a tortuous niche inside the bone inhabited by osteocytes which comprise >90% of the bone cells and regulate the bone homeostasis (Figure 2G) (Genthial et al., 2017, 2019; Tokarz et al., 2017), and the myelinated fibers in the cerebral cortex (Figure 2H) (Redlich and Lim, 2019) which are of significance in the MS pathogenesis (Peterson et al., 2001; Bo et al., 2003; Calabrese et al., 2010). The function of these structures depends on the 3D connectivity which THG can acquire in entirety while reflectance-based imaging omits the axial population. Therefore, THG is better suited for unraveling the morphological dynamics and the plasticity of the meshwork.

### Toward Exogenous THG Agents

The development of exogenous THG agents has been exploratory to date. Contrary to SHG, to design molecules with large susceptibility has not been a major avenue of development because the refractive index mismatch within the focal volume, thus the geometrical factors, is far more essential for THG imaging. Instead, a successful approach pertinent to intravital studies has been to enhance the THG contrast by way of a near-resonance excitation of organic aggregates, such as porphyrin. Since an initial demonstration of THG enhancement by the absorption band of hemoglobin (Clay et al., 2006), packaged porphyrins have been proved as a THG agent (Cui et al., 2015). Also, the hemozoin pigment, a natural crystalline aggregate of hemes produced by parasites, emits a strong THG signal which can be exploited for detecting malaria (Belisle et al., 2008; Tripathy et al., 2013). However, given its famous role as a generator of singlet oxygen, porphyrin-based THG imaging raises a concern of photosafety. Nevertheless, the strategy of resonantly enhancing THG is applicable to any aggregate of molecules with distinct absorption bands. For native molecules, which tend to possess broad spectra, a tunable light source might be necessary for optimization.

## Conclusion: Future Prospects

We have introduced recent tricks of optical harmonic generation which allownovel information to be extracted about the CNS of living animals. Innovations in deep-tissue microscopy have motivated ingenious intravital preparations for observing the intra- and intercellular dynamics beyond 2D cultured cells, thus opening new fields of investigation. Similar synergy is expected to follow the empowered intravital SHG and THG microscopy, enabling novel discoveries in neuroscience.

## Author Contributions

The author confirms being the sole contributor of this work and has approved it for publication.

### Conflict of Interest

The author declares that the research was conducted in the absence of any commercial or financial relationships that could be construed as a potential conflict of interest.
